# Functional Characterization of *Clostridium tyrobutyricum* L319: A Promising Next-Generation Probiotic for Short-Chain Fatty Acid Production

**DOI:** 10.3389/fmicb.2022.926710

**Published:** 2022-06-17

**Authors:** Zhihan Yang, Fatima Ezzahra Amal, Lei Yang, Yuxin Liu, Liying Zhu, Zhengming Zhu, Ling Jiang

**Affiliations:** ^1^College of Biotechnology and Pharmaceutical Engineering, Nanjing Tech University, Nanjing, China; ^2^College of Food Science and Light Industry, Nanjing Tech University, Nanjing, China; ^3^College of Chemical and Molecular Engineering, Nanjing Tech University, Nanjing, China; ^4^State Key Laboratory of Materials-Oriented Chemical Engineering, Nanjing Tech University, Nanjing, China

**Keywords:** *Clostridium tyrobutyricum*, safety evaluation, probiotics, prebiotics, short-chain fatty acids

## Abstract

Probiotics contribute a lot to human health and the occurrence of diseases. Correspondingly, probiotics’ safety evaluation and probiotic properties have received increasing attention in the food industry and disease treatment. *Clostridium tyrobutyricum* L319 is a short-chain fatty acid (SCFA)-producing strain isolated from Grana Padano cheese with a blowing defect. Our previous study has shown its safety at the genomic level. This study focused more on the safety evaluation and probiotic properties *in vitro*. According to the results, this strain has no potential virulence factors or the possibility of antibiotic resistance genes propagation. It also fulfilled several criteria to be used as a probiotic, including significant hydrophobicity under an acidic condition (pH 5.0) and resistance to simulate gastric juice and intestinal juice. Additionally, this strain was found to be tolerant to the harsh conditions of the external environment, including resistance to low (20°C) and high (50°C) temperatures, high salts (3% NaCl), and low pH (pH 5.0). Finally, we found that this strain could ferment prebiotics, such as chito-oligosaccharides, to produce SCFAs. It exhibited excellent growth performance whether using chito-oligosaccharide as a sole carbon source or combining glucose as the mixed carbon source. Furthermore, chito-oligosaccharide and glucose (1:1) mixed carbon sources were the optimal strategy for the production of SCFAs. Our findings demonstrated that this strain might be considered a promising candidate for future use as a probiotic to promote health benefits.

## Introduction

The gut microbiota is rich in diversity, including bacteria, viruses, fungi, and protozoa (Joukar et al., 2020). Among them, the number of bacteria alone is as high as 100 trillion; therefore, the gut microbiome is also called the second genome of human beings (Zhu et al., 2010). With the development of metagenomic sequencing and bioinformatics technology, various studies have shown an inseparable relationship between gut microbes and the host (Shreiner et al., 2015). The host’s balance of gut microbiota is mainly regulated through diet (Valdes et al., 2018). The gut microbiota obtains energy from the undecomposed substances in the gut for their growth and participates in the substance metabolism, nutrient absorption, and physiological and immune defense processes (Elzinga et al., 2019). Moreover, prebiotics is a class of substrates that can be selectively utilized by gut microbiota and are beneficial to health, including fructo-oligosaccharides, galacto-oligosaccharides, and inulin (Sonnenburg and Sonnenburg, 2019). They could enrich specific microorganisms and produce metabolites, such as short-chain fatty acids (SCFAs), that could contribute to human health (Bindels et al., 2015).

Short-chain fatty acids are the main products of intestinal microbial fermentation. Intestinal bacteria could produce SCFAs by using dietary fibers as substrates. The glycoside hydrolase produced by bacteria could convert dietary fibers into monosaccharides and then generate the main end-product SCFAs through anaerobic fermentation (Xu et al., 2020). SCFAs are a group of carboxylic acids containing six or fewer carbon molecules, particularly acetate, propionate, and butyrate. Approximately, 500–600 mmol of SCFAs in the gastrointestinal tract are produced per day, depending on dietary fiber content (Blaak et al., 2020). Moreover, acetate, propionate, and butyrate were produced in an overall molar proportion of ∼60:20:20, respectively, representing 95% of SCFAs in the human colon (Zhu et al., 2022). Numerous studies have shown that SCFAs are essential for maintaining the metabolic function of the host. They could provide energy for intestinal epithelial cells, protect the intestinal barrier, and regulate intestinal inflammation (Hu et al., 2018). Furthermore, among the SCFAs, butyrate has been investigated extensively and is the primary energy source for colon cells (Wang et al., 2020). It was found that the gut butyrate-producing bacteria mainly belong to phylum Firmicutes and can be further subdivided into the genus *Clostridium* (Xu et al., 2020).

At present, many studies are focusing on the health effects of *Clostridium* and its metabolite SCFAs. A mixture of human gut-derived 17 *Clostridium* strains was applied in a previous study to investigate the effects of preventing and treating multiple sclerosis and reveal the mediating role of their metabolite butyrate (Calvo-Barreiro et al., 2021). Also, *Clostridium butyricum*, a butyrate-producing probiotic, was found to increase SCFA levels, thereby inhibiting high-fat diet-induced intestinal tumor formation (Chen D. et al., 2020). Given the importance of *Clostridium*, it is necessary to evaluate its safety and probiotic properties. By now, only *C. butyricum* CBM588 was authorized as a novel probiotic for use by humans and food animals according to Regulation (EC) No. 258/97 (Isa et al., 2016). In addition, a novel *Faecalibacterium prausnitzii* strain was validated *in vitro* to identify its potential as a next-generation probiotic (Martín et al., 2017). Our previous study found that *C. tyrobutyricum*, a Gram-positive, acidogenic, anaerobic bacterium, could produce butyrate as the main fermentation product and acetate as co-products. It is the best strain reported for butyrate production (Jiang et al., 2018).

Meanwhile, *C. tyrobutyricum* can be used in the treatment and/or prevention of certain human and animal diseases. Recent studies have revealed that *C. tyrobutyricum* ATCC25755 could protect against lipopolysaccharide (LPS)-induced epithelial dysfunction and inflammation (Xiao et al., 2021b,c). Also, as a potential probiotic, it could alleviate *Staphylococcus aureus*-induced endometritis in mice by inhibiting endometrial barrier disruption and inflammatory response (Hu et al., 2019).

At present, there is an increasing interest in applying gut microbes as potential probiotic agents (Zhang et al., 2021). Due to safety concerns, lack of safety information, and legislation, only a limited number of *Clostridium* species are commercialized. Although little is known about the safety of *C. tyrobutyricum*, there is a clear promise for this species as a next-generation probiotic. Our previous study conducted a genome sequence analysis of *C. tyrobutyricum* L319 and evaluated its safety at the genomic level. The results indicated that this strain does not possess transferable resistance genes, invasive defensive pathogenicity factors, or harmful enzymes (Liu et al., 2020). In this study, as recommended (FAO/WHO) (Araya et al., 2002), phenotypic testing, prebiotics utilization, metabolite production, biochemical activity, and toxicity testing, in combination with genetic analysis, were performed to validate whether *C. tyrobutyricum* L319 is safe for use as a next-generation probiotic in the food industry and gut therapy.

## Materials and Methods

### Bacterial Strains and Culture Conditions

*Clostridium tyrobutyricum* L319 used in this study was previously isolated from Grana Padano cheese with a blowing defect (Shanghai Rongyue Trading Co., Ltd., Shanghai, China). The genome was previously isolated drafted and analyzed in our previous study (Liu et al., 2020). *C. tyrobutyricum* was cultured at 37°C in reinforced clostridial medium (RCM), containing (per liter) 10 g tryptone, 10 g beef extract, 5 g glucose, 5 g NaCl, 3 g yeast extract, 3 g anhydrous sodium acetate, 1 g soluble starch, and 0.5 g L-cysteine, in serum bottles with a final concentration of 0.05% as an oxygen indicator under strictly anaerobic condition. The fermentation was carried out in a TGY medium containing (per liter) 30 g tryptone, 20 g glucose, 10 g yeast extract, and 1.3 g L-cysteine. In addition, the minimal medium used to evaluate the effects of different prebiotics containing (per liter) 1 g NH_4_Cl, 0.1 g KCl, 0.2 g MgSO_4_⋅7H_2_O, 0.8 g NaCl, 0.1 g KH_2_PO_4_, 20 mg CaCl_2_⋅2H_2_O, trace elements (per liter: 0.10 g ZnSO_4_⋅7H_2_O, 0.03 g MnCl_2_⋅4H_2_O, 0.30 g H_3_BO_3_, 0.20 g NiCl_2_⋅6H_2_O, 0.01 g CuCl_2_2H_2_O, 0.02 g NiCl_2_⋅6H_2_O, 0.03 mg Na_2_MoO_4_⋅2H_2_O), and Wolfe’s vitamin solution (per liter: 20 mg biotin, 20 mg folate, 100 mg pyridoxine hydrochloride, 50 mg thiamine hydrochloride, 50 mg riboflavin, 50 mg nicotinic acid, 50 mg calcium pantothenate, 1 mg vitamin B_12_, 50 mg 4-aminobenzoic acid, 50 mg lipoic acid). All media were sterilized by autoclaving at 108°C for 30 min.

### Hierarchical Cluster Analysis

The neighbor-joining phylogenetic tree was constructed based on 16S rRNA gene sequences of the representative *C. tyrobutyricum* and its neighbors, *C. butyricum.* The average nucleotide identity (ANI) was calculated using OrthoANI (Lee et al., 2016). The evolutionary analysis of gene synteny and collinearity was conducted with MCScanX in the TBtools box (Wang et al., 2012; Chen C. et al., 2020).

### Antibiotic Resistance Testing

Antibiotic resistance testing was performed using the minimum inhibitory concentration (MIC) method described in a previous study (Baccouri et al., 2019). The tested antibiotics were gentamicin, kanamycin, streptomycin, clindamycin, chloramphenicol, tetracycline, vancomycin, erythromycin, neomycin, ampicillin, linezolid, trimethoprim, rifampicin, and ciprofloxacin. The overnight cultures of bacteria were diluted with RCM medium to 10^5^–10^6^ CFU ml^–1^. Then, the antibiotic solutions and bacterial suspension were added to a 96-well plate from low to high concentrations, according to Supplementary Table 1. In addition, the one that contains the RCM medium and the bacterial suspension was considered the positive control. In contrast, others with RCM medium and phosphate-buffered saline (PBS) were negative control. MIC was determined by measuring optical density at 600 nm (OD_600_) with a microplate reader (SpectraMax, M2). The test was performed in triplicates. The MIC breakpoint is the concentration where no growth is exhibited. The sensitivity/resistance was confirmed regarding the microbiological cutoff values prescribed by the European Food Safety Authority (EFSA) in 2012 guidelines (ESFA, 2012). Strains exhibiting MIC values less than the breakpoints were considered sensitive. Otherwise, they were resistant.

### PCR Amplification of Virulence Genes and Biogenic Amine Genes

According to the NCBI database, the neurotoxin genes of types A, B, E, and F from *Clostridium botulinum* [GenBank were A (EU429475.1), B (M81186.1), E (AB082519.1), F (M92906.1)] and alpha, beta, epsilon, iota from *C. perfringens* [alpha (L43546.1), beta (X83275.1), and epsilon (M95206.1), iota (X73562.1)] were selected to be detected on the genome of *C. tyrobutyricum* L319 by PCR reactions. Genes coding amino acid decarboxylases, HDC1 and HDC2 (both related to histidine decarboxylase), tyrosine decarboxylase (TDC), ornithine decarboxylase (ODC), and agmatine deaminase (AGDI) were also detected. The amplified products were separated by electrophoresis on 2% (w/v) agarose gels in 0.5 × TAE buffer. Gels were stained in TAE buffer containing 0.5 mg⋅ml^–1^ ethidium bromide. All the primers used are listed in Supplementary Table 2.

### Hemolytic Assay

The overnight cultures of bacteria were diluted to 10^–6^ CFU with a 10-fold gradient of sterile PBS, and 100 μl of the bacterial suspension diluted to 10^–6^ was coated on the blood AGAR plate. Two parallel experiments were performed after anaerobic culture for 48 h, and two parallel experiments were performed to observe the hemolysis rings around the bacterial colonies on the blood agar plate.

### Assessment of Potential Probiotic Properties

#### Hydrophobicity Assay

To evaluate cell surface hydrophobicity at different pH values, the strain was collected after overnight cultivation by centrifugation (6,000 × *g* for 5 min at 4°C), washed twice with PBS (50 mM, pH 5, pH 6, pH 7) and then resuspended in the same buffer that was adjusted until A560 values (A_0_) around 1. In addition, *N*-hexadecane was then added five times the volume to the cell suspension (1:5) and adequately mixed for 120 s. The aqueous layer’s A560 value (A) was measured after 1 h at 37°C. Then, the hydrophobicity was calculated by the equation: %H = [(A_0_ − A)/A_0_] × 100.

#### Tolerance to the Simulated Gastrointestinal Tract

The simulated intestinal juice contains 1% (w/v) pancreatin, 0.3% (w/v) bile salt, and was adjusted to pH 6.8, while the simulated gastric juice contains 3% (w/v) pepsin, 2% (w/v) NaCl, and was adjusted to pH 3.0. The strain was collected with the same method in hydrophobicity assay and 100 μl of the bacterial suspension was diluted to 10^–6^ coated on the RCM agar plate, considered as CFU_0_. Then, the remaining strains were divided into two groups (25 ml for each), separately growing in the intestinal juice for 4 h and gastric juice for 2 h (100 r/min, 37°C) with strains growing in PBS as control. The above steps were repeated to coat the bacterial suspension on the RCM agar plates. The colonies on the plates were counted after growing on the plates for 24–48 h, the CFUs were calculated, and the tolerance was evaluated by the equation: %T = [log_10_CFU/ml]/[log_10_CFU_0_/ml] × 100.

#### Temperature, NaCl, and Acid Tolerance

To assess the robustness of *C. tyrobutyricum* L319 at several temperatures, the overnight cultures were inoculated in 30 ml fresh RCM medium at 1% (v/v) with different temperatures (4, 20, 30, 37, 40, 45, and 50°C); NaCl (0, 1, 2, 3, and 4%); and pH (4.5, 5.0, and 6.5). The growth profiles were measured at OD_600_ at intervals. The expected growth conditions at 37°C, pH 6.5, and 0% NaCl were set as the control group. The growth experiments were performed in triplicate.

#### Fermentation Kinetics of Short-Chain Fatty Acids Under Acidic Conditions

The overnight cultures were transferred to a 50 ml fresh TGY medium with a volume ratio of 5% at pH 7.0 and 5.0, respectively. The medium at pH 5.0 was adjusted with butyrate. The cell growth, butyrate, and acetate production were determined.

#### Growth Profiling in Different Prebiotics Containing Media

A total of eight prebiotics, including fructo-oligosaccharide, chitosan oligosaccharide, galacto-oligosaccharide, xylo-oligosaccharide, isomalto-oligosaccharide, inulin, raffinose, and lactitol, were used to determine the growth profiling of *C. tyrobutyricum* L319. Each prebiotic was the same C-mole as glucose in a minimal medium. The control group was the minimal medium adding 10 g⋅L^–1^ glucose. The other components in the medium were the same as the minimal medium. Strains were cultivated in serum bottles, while growth performances were determined at OD_600_. In addition, to investigate the growth profiling of *C. tyrobutyricum* L319 with glucose and prebiotics as mixed carbon sources, every prebiotic was the same C-mole as glucose (5 g⋅L^–1^ glucose) in the minimal medium that also contained 5 g⋅L^–1^ glucose. Strains were also cultivated in serum bottles, and the growth performances were determined at OD_600_. Lastly, the different ratios of chitosan oligosaccharide and glucose (1:1; 1:2; 1:3; 2:1, 3:1 C-mole) were added into the minimal medium, and the growth performances were determined.

#### Analytical Methods

Cell growth was monitored by measuring the optical density at 600 nm using a spectrophotometer (Shanghai Spectrum, China). The glucose, butyrate, and acetate concentrations were assessed using high-performance liquid chromatography (HPLC, LC-15C; Shimadzu) equipped with an organic acid analysis column (HPX-87H, Bio-Rad) at 65°C and with 0.005 M sulfuric acid as the mobile phase at 0.6 ml⋅min^–1^. HPLC also analyzed chito-oligosaccharide with a Carbonmix Ca-NP column (Sepax Technologies, Suzhou, China) kept at 85°C. Water was also used as the mobile phase with a 0.6 ml⋅min^–1^ flow rate.

## Results

### Hierarchical Cluster Analysis Between *Clostridium tyrobutyricum* and *Clostridium butyricum*

**FIGURE 1:**
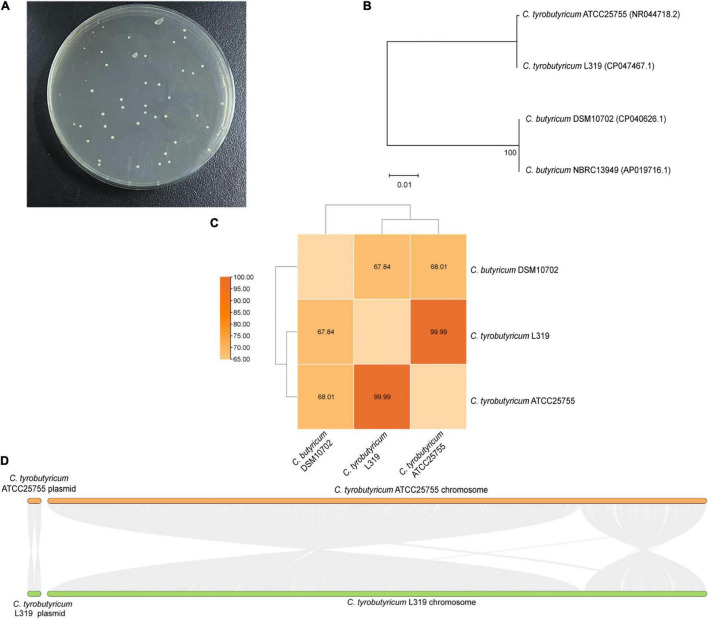
**(A)** The growth of *C. tyrobutyricum* L319 on RCM plates. **(B)** The neighbor-joining phylogenetic tree based on the 16S rRNA gene sequences. **(C)** Heatmap of ANI between *C. tyrobutyricum* L319, *C. tyrobutyricum* ATCC 25755, and *C. butyricum* DSM10702. ANI values (%) are indicated. **(D)** Pairwise alignment between the reference strain *C. tyrobutyricum* ATCC 25755 and the related strain *C. tyrobutyricum* L319.

The *C. tyrobutyricum* strain was isolated from Grana Padano cheese with a blowing defect. Moreover, *C. butyricum* was authorized as a novel ingredient according to Regulation (EC) No. 258/97. Therefore, in this study, we investigated the similarity of *C. tyrobutyricum* and the reference probiotic strain *C. butyricum*. The colony shapes of these two strains were relatively close, and it is not easy to find a significant difference (Figure 1A). Besides identifying colonies, a phylogenetic tree was constructed based on the 16S rRNA sequences from evolutionary distances by the neighbor-joining method. Analysis of the phylogenetic tree depicted the hierarchical relationship between the four strains and suggested that *C. tyrobutyricum* L319 was closely related to the potential probiotic *C. tyrobutyricum* ATCC25755 (Figure 1B). Whole-genome analysis using the ANI also exhibited that *C. tyrobutyricum* L319 is closer to *C. tyrobutyricum* ATCC25755 (ANI > 95%) in terms of genomic distance (Figure 1C). Finally, the overall genomic differences between *C. tyrobutyricum* L319 and *C. tyrobutyricum* ATCC25755 were analyzed by aligning the two genomes using MCScanX. The results suggested a high level of conservation along the chromosome and plasmid. Indeed, these two genomes were found to be organized in a very similar way (Figure 1D). These results indicated that *C. tyrobutyricum* L319 has a certain identity with the reference probiotic *C. butyricum* DSM10702 and has a high degree of homology with the potential probiotic *C. tyrobutyricum* ATCC25755 at the genome level.

### Safety Evaluation of *Clostridium tyrobutyricum* L319

#### Assessment of the Antibiotic Resistance

The antibiotic resistance of *C. tyrobutyricum* L319 against 14 tested antibiotics is shown in Table 1. Generally, strains were considered resistant when the MIC values were higher than breakpoint values according to the EFSA. Overall, this strain showed the ability to resist the impact of aminoglycoside antibiotics (gentamicin, kanamycin, and streptomycin), clindamycin, and chloramphenicol. In contrast, this strain was susceptible to tetracycline, vancomycin, and erythromycin, which agrees with the antibiotic resistance analysis for *C. butyricum* CBM5881 (Isa et al., 2016). In addition, the breakpoints of other antibiotics, including neomycin, ampicillin, linezolid, trimethoprim, and rifampicin, were not established by the EFSA, the strain was also considered susceptible to neomycin (MIC = 0.5 μg⋅ml^–1^), ampicillin (MIC = 0.032 μg⋅ml^–1^), linezolid (MIC = 0.032 μg⋅ml^–1^), trimethoprim (MIC = 0.125 μg⋅ml^–1^), rifampicin (MIC = 0.125 μg⋅ml^–1^), and ciprofloxacin (MIC = 8 μg⋅ml^–1^) according to the results for antibiotic susceptibility of *C. butyricum* CBM5881 (Isa et al., 2016).

**TABLE 1:** Antibiotic resistance profiles of *C. tyrobutyricum* L319.

Antibiotics	Breakpoint μg/ml	MIC μg/ml	Resistance or sensitivity
Gentamicin	4	>256	R
Kanamycin	8	128	R
Streptomycin	8	16	R
Clindamycin	4	>16	R
Chloramphenicol	8	16	R
Tetracycline	8	0.25	S
Vancomycin	4	0.25	S
Erythromycin	4	4	S
Neomycin	NA	0.5	S
Ampicillin	NA	0.032	S
Linezolid	NA	0.032	S
Trimethoprim	NA	0.125	S
Rifampicin	NA	0.125	S
Ciprofloxacin	NA	8	S

*NA, not available; S, sensitivity; R, resistance.*

#### Characterization of Virulence Potential

Identifying virulence genes in *C. tyrobutyricum* L319 by molecular and phenotypic procedures is necessary to avoid the risk of dissemination of virulence genes to other bacteria by genetic transfer. In our previous study, the genome of *C. tyrobutyricum* L319 was analyzed to determine the presence of neurotoxin genes (types A, B, E, and F) and toxin genes (alpha, beta, epsilon, and iota). Meanwhile, genes associated with biogenic amine biosynthesis, such as HDC1 and HDC2, TDC, ODC, and AGDI were also analyzed. Fortunately, these genes were not found in *C. tyrobutyricum* L319 by whole-genome sequencing (Liu et al., 2020). We checked these genes by PCR assays in this study, and the 16S rRNA gene was amplified as the control group (Supplementary Figure 1). The results showed that the neurotoxin and toxin genes were not detected in *C. tyrobutyricum* L319, and none of the genes associated with biogenic amine biosynthesis were detected.

**FIGURE 2:**
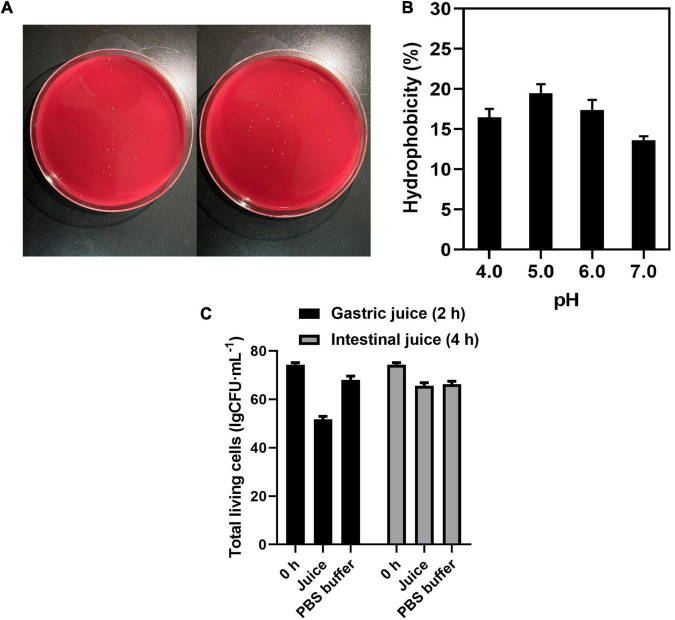
**(A)** Hemolysis of *C. tyrobutyricum* L319 on blood plate. **(B)** Hydrophobicity assay of *C. tyrobutyricum* L319 at pH 4.0, 5.0, 6.0, and 7.0. **(C)** The total living cells survive in the gastric juice and intestinal juice. Controls all refer to the strains suspended in PBS.

To develop new beneficial microbes, their absence of hemolysis capacity should be evaluated. As mentioned above, hemolytic activity has been determined on the blood AGAR plate. Observation of the blood AGAR plates showed no α-hemolysis and β-hemolysis activities, while the strain showed γ-hemolysis activity, known as negative or no hemolysis activity (Figure 2A).

### Probiotic Potential of *Clostridium tyrobutyricum* L319

#### Hydrophobicity and Simulated Intestinal Juice and Gastric Juice Tolerance

Cell surface hydrophobicity is a beneficial attribute for probiotics, and it is a non-specific interaction between microbial cells and the host. Therefore, the affinity of *C. tyrobutyricum* L319 toward the hydrophobic solvent *N*-hexadecane was investigated. The results of this experiment showed less than 20% hydrophobicity. However, it could be found that the strain presented the highest hydrophobicity at pH 5.0 (19.13%) when compared with that at pH 4.0 (16.23%), 6.0 (17.53%), and 7.0 (13.14%) (Figure 2B). It could be indicated that the strain exhibited better hydrophobicity under the pH conditions between 5.0 and 6.0.

The tolerance toward simulated intestinal juice and gastric juice is the essential characteristic for the strain to colonize the intestine. Our study indicated that *C. tyrobutyricum* L319 has a promising tolerance to gastric juice and intestinal juice with a survival ratio of 69.67% (2 h) and 88.27% (4 h) (Figure 2C), compared with other potential probiotic products (<56%) (Supplementary Table 3).

#### Temperature, NaCl, and Acid Tolerance

**FIGURE 3:**
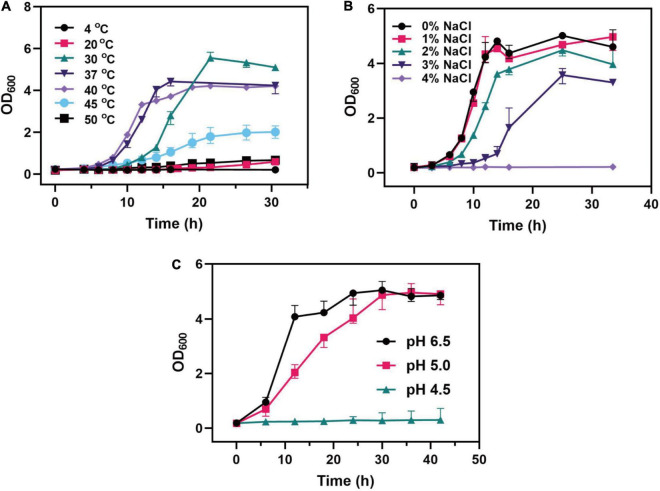
Effects of **(A)** different temperatures, **(B)** NaCl concentrations, and **(C)** pH values on the growth of the *C. tyrobutyricum* L319 strain.

Probiotics are inevitably faced with various stresses from the external environment in the fermentation products and the human gastrointestinal tract, such as heat stress caused by high temperature and osmotic stress caused by water loss during the spray drying process of bacterial agents. Also, probiotics suffer gastric acid stress in the gastrointestinal tract. This stress seriously affected the growth, metabolism, and physiological functions of probiotics. Therefore, *C. tyrobutyricum* L319 was tested for its ability to survive in acidic conditions, low- and high-temperature conditions, and in the presence of NaCl. It could be found that *C. tyrobutyricum* L319 had different tolerance in different environments in this study (Figure 3). The growth profiling of *C. tyrobutyricum* L319 was slightly affected by temperature, and *C. tyrobutyricum* L319 displayed excellent tolerance to low and high temperatures, ranging from 20 to 50°C (Figure 3A). The maximum cell density at 30°C was 5.6; however, the lag period was longer than 37 and 40°C. The optimum growth temperature of 37°C was taken as the dividing line. With the culture temperature rising and falling, the maximum biomass was lower, and the lag period was longer. In addition, it can be found that the strain could not grow at 4°C but could still grow at 50°C, indicating that the strain has excellent tolerance to high temperatures.

Regarding the tolerance toward the osmotic pressure, *C. tyrobutyricum* L319 could survive under the 3% NaCl condition, and the maximum cell density reached about 31.5% of the density in the 0% NaCl condition. It was found that no significant variability was exhibited under the 1% NaCl condition. However, the strain could not grow under the 4% NaCl condition (Figure 3B). The viability of *C. tyrobutyricum* L319 was also assessed under the acidic condition, incubated in RCM broth supplemented with butyrate. Our findings indicated that the strain exhibited high tolerance to acidity after exposure to media pH 5.0. The maximum OD_600_ of *C. tyrobutyricum* L319 at pH 5.0 was close to the control at pH 6.5 (OD_600max_: 4.99 vs. 5.16). However, it exhibited no growth at pH 4.5.

#### Fermentation of Short-Chain Fatty Acids

**FIGURE 4:**
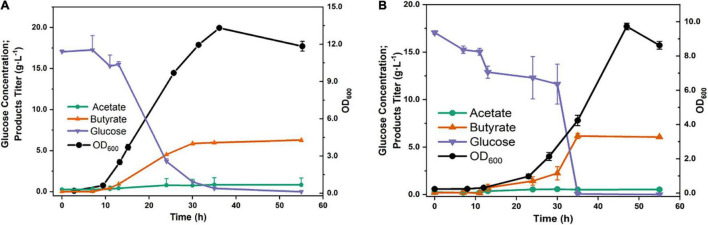
Fermentation products of strain under natural (pH 6.5) **(A)** and lower (pH 5.0) **(B)** pH values.

In general, *C. tyrobutyricum* has already been considered a promising microbial host for the production of SCFAs, especially butyrate. Due to its unique butyrate synthetic pathway, *C. tyrobutyricum* mainly produces butyrate and acetate. Therefore, the batch fermentation results showed that the titer of butyrate was 6.29 g⋅L^–1^, when fed with 20 g⋅L^–1^ glucose, the yield was considerably achieved at 0.31 g⋅g^–1^. Furthermore, a much lower acetate titer was detected than butyrate (0.85 vs. 6.29 g⋅L^–1^) with a yield of 0.04 g⋅g^–1^ (Figure 4A). In addition, the fermentation of SCFAs at pH 5.0, which was close to the intestinal environment, was also investigated. Interestingly, the titer of butyrate was not significantly decreased (6.16 vs. 6.29 g⋅L^–1^) (Figure 4B). However, the proportion of butyrate/acetate came to 11, higher than that at pH 6.5 condition (BA/AA = 7.4). The results indicated that *C. tyrobutyricum* L319 has a great potential to be applied in the production of SCFA, which has already been recognized as a major product of intestinal microbial fermentation with beneficial effects on human health.

#### The Growth Profiling and Production of Short-Chain Fatty Acids With Different Prebiotics

**FIGURE 5:**
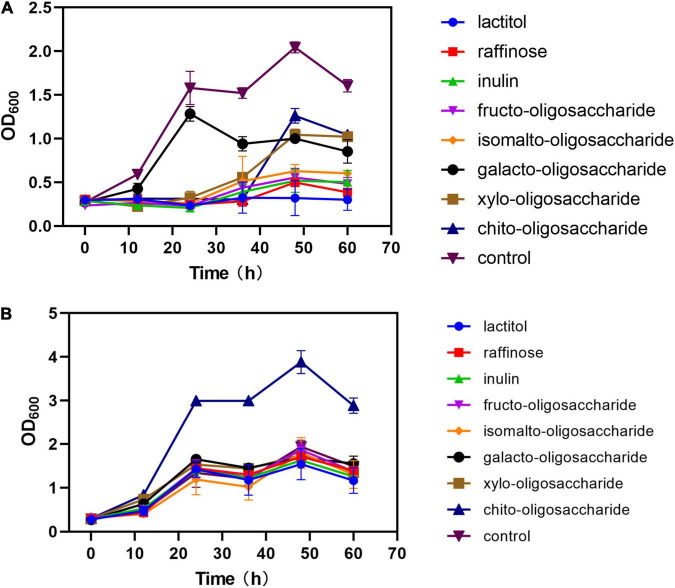
Effects of different prebiotics added on the growth of strain. **(A)** Prebiotics were added as sole carbon source in the same C-mole of 10 g⋅L^– 1^ glucose. **(B)** Prebiotics were added to the media with 5 g⋅L^– 1^ glucose (1:1 C-mole). Control in panels **(A,B)** refers to 10 g⋅L^– 1^ glucose as sole carbon source.

When the eight prebiotics were used separately as the sole carbon source, most of the strains did not exhibit growth compared with glucose as control, except for those media using galacto-oligosaccharide (OD_600_max: 1.29 vs. 2.05), xylo-oligosaccharide (OD_600_max: 1.04 vs. 2.05), and chito-oligosaccharide (OD_600_max: 1.26 vs. 2.05) (Figure 5A). Also, the strains growing in galacto-oligosaccharide showed a similar lag phase to those in glucose, while the strains in xylo-oligosaccharide and chito-oligosaccharide spent about four times longer adjusting to the conditions. In addition, prebiotics was also detected as complimentary carbon source separately added to an optimized medium containing 10 g⋅L^–1^ glucose with the same C-mole volume. The results (Figure 5B) showed that the strains growing in mixed carbon sources of glucose and chito-oligosaccharide exhibited outstanding biomass, even compared with glucose (3.88 vs. 1.94) as the sole carbon source. However, there are no significant differences found in other optimized media.

**FIGURE 6:**
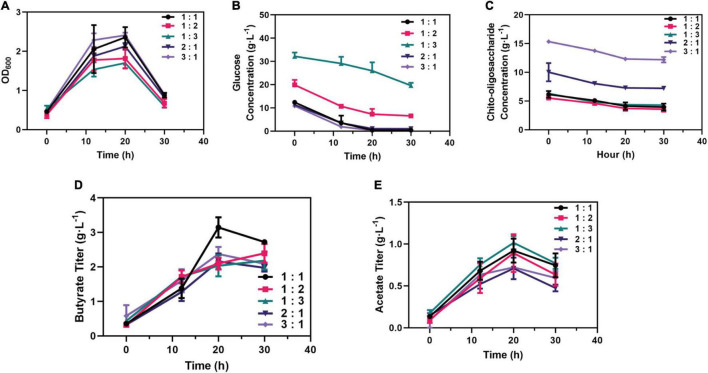
Optimizing added strategy of the proportion (C-mole) of chito-oligosaccharide and glucose. **(A)** Growth performance of strains in medium supplemented with different proportions of carbon sources. **(B)** Consumption of glucose in medium supplemented with different proportions of carbon sources. **(C)** Consumption of chito-oligosaccharide in medium supplemented with different proportions of carbon sources. **(D)** Production of butyrate supplemented with different proportions of carbon sources. **(E)** Production of acetate supplemented with different proportions of carbon sources.

Further, the different ratios of chito-oligosaccharide and glucose as mixed carbon sources were investigated; the results (Figure 6A) showed that the strains growing in the mixed carbon sources of 3:1 C-mole reached the highest growth rate (μ = 0.06 h^–1^), followed by the ratio of 1 (μ = 0.05 h^–1^). Interestingly, when the same concentration of chito-oligosaccharide was added in the media. Commensurately, among the strains (Figure 6B), the strains in medium (3:1 C-mole) exhibited the highest glucose consumption (0.37 g⋅L^–1^⋅h^–1^) while the lowest (0.13 g⋅L^–1^⋅h^–1^) was obtained in the ratio of 1:3. In terms of the consumption of chito-oligosaccharide, strains in the medium of 3:1 C-mole reached the highest rate (0.15 g⋅L^–1^⋅h^–1^). However, the chito-oligosaccharides in all media were only consumed about 2 g⋅L^–1^ (Figure 6C).

In addition, the production of SCFAs in each medium was also investigated, which showed that the medium containing chito-oligosaccharide and glucose with 1:1 C-mole reached the outstanding titer of butyrate (3.15 g⋅L^–1^) and the yield of 0.31 g⋅g^–1^ from glucose and 0.50 g⋅g^–1^ from chito-oligosaccharide (Figure 6D). However, the highest titer of acetate (1.01 g⋅L^–1^, a yield of 0.03 g⋅g^–1^ from glucose consumed and 0.20 g⋅g^–1^ from chito-oligosaccharide) was obtained in the medium of 1:3 C-mole (Figure 6E), which might be related to the need to maintain the growth according to the growth profile in Figure 6A. Besides, the strains in the medium of 1:1 C-mole could also reach 0.92 g⋅L^–1^ in titer and yield of 0.09 g⋅g^–1^. Overall, the mixed carbon sources with 1:1 C-mole of chito-oligosaccharide and glucose were the optimal ratio for the production of SCFAs.

## Discussion

Probiotics are referred to as non-pathogenic live microorganisms and can confer health benefits on the host (Araya et al., 2002). Due to the potential for infection or toxin production, probiotics should meet health and safety requirements before commercial application. The primary responsibility for certifying a probiotic is determining its safety as any adverse effects should be anticipated ahead of time. Generally, biochemical characterization, risk assessment, and probiotic properties are critical components in evaluating a new probiotic (Oh and Jung, 2015). Previously, we demonstrated that the strain *C. tyrobutyricum* L319, isolated from the Grana Padano cheese with a blowing defect, is more competitive than isolates from other environments, exhibiting safety on the genome level (Liu et al., 2020). Although the health benefits of *C. tyrobutyricum* are well-acknowledged, considerable study remains to be done before this species can be certified as a probiotic. Therefore, we performed a more detailed characterization and systemic evaluation of *C. tyrobutyricum* L319 in this study, and we suggest that non-toxic *C. tyrobutyricum* is an excellent probiotic candidate.

Generally, *C. butyricum* was included in the list of microorganisms that have been developed as a probiotic for use in the food according to Regulation (EC) No. 258/97 (Isa et al., 2016). Meanwhile, previous studies have shown that a potential probiotic *C. tyrobutyricum* ATCC25755 could protect against LPS-induced colonic inflammation and epithelial dysfunction (Xiao et al., 2021a,c). Therefore, we investigated the similarity between the reference probiotic strain *C. butyricum* and *C. tyrobutyricum*, including identification of colonies, phylogenetic tree analysis, ANI analysis, and evolutionary analysis of gene synteny and collinearity. These results showed that *C. tyrobutyricum* L319 has a high degree of homology with the probiotic *C. tyrobutyricum* ATCC25755 at the genome level (Figure 1). Combined with the safety evaluation of *C. tyrobutyricum* at the genomic level in our previous study and its high homology and conservation with another *C. tyrobutyricum*, we can conclude that this bacterium exhibits a good safety profiling at the genomic level.

Except for the safety evaluation at the genomic level, antibiotic resistance, virulence-related assay, and hemolytic activity were all carried out. First, we verified the antibiotic resistance phenotype of *C. tyrobutyricum* L319 and found that it is resistant to gentamicin, kanamycin, streptomycin, clindamycin, and chloramphenicol but susceptible to tetracycline, vancomycin, erythromycin, neomycin, ampicillin, linezolid, trimethoprim, rifampicin, and ciprofloxacin (Table 1). The MICs of these antibiotics for *C. tyrobutyricum* L319 were also identified. Interestingly, we found that the vancomycin resistance exhibited discrepancies between the genotype and observed phenotype (Liu et al., 2020), which could be because potential antibiotic resistance genes were annotated based on 40% amino acid homology, which means that this gene may be not present in the genome of *C. tyrobutyricum* L319. Similarly, aminoglycoside (gentamicin, kanamycin, and streptomycin) resistance in *C. tyrobutyricum* L319 was also described as innate resistance in other *C. butyricum* strains, meaning that aminoglycoside resistance is not a specific trait of *C. tyrobutyricum* L319 (Isa et al., 2016). In addition, there is no safety hazard that all the genes associated with antibiotic resistance were found unable to be transferred into the environment according to our previous study (Liu et al., 2020). Furthermore, the absence of virulence-related genes is a desired feature for *C. tyrobutyricum* employed in the food industry.

Our previous study identified 30 potential virulence factors in the *C. tyrobutyricum* L319 genome. Fortunately, none of these genes need to be excluded according to EFSA guidelines (Liu et al., 2020). Meanwhile, the neurotoxin and toxin genes were not identified, and none of the genes associated with biogenic amines biosynthesis were also detected (Supplementary Figure 1). In addition, its hemolytic activity was confirmed by hemolysis tests, and the strain *C. tyrobutyricum* L319 exhibited no hemolytic activity (Figure 2A).

We also assessed the potential probiotic properties for the strain *C. tyrobutyricum* L319. Hydrophobicity is one of the suggestive parameters for cell surface properties of probiotics, and it is closely related to the adhesion ability of probiotics (Zuo et al., 2016). Previously, probiotics’ ability to attach to epithelial cells and promote health benefits increased as their hydrophobicity increased (Nami et al., 2019). In this study, *C. tyrobutyricum* L319 exhibited better hydrophobicity percentages under the conditions between pH 5.0 and 6.0 (Figure 2B) and an excellent tolerance toward simulated gastric juice and intestinal juice (Figure 2C), which exhibits a possibility for this strain to colonize the intestine.

In our results, *C. tyrobutyricum* L319 exhibited higher anti-stress tolerance (Figure 3). The growth of the strain was stimulated under different temperatures and NaCl concentrations. We found that the strain displayed excellent tolerance to low and high temperatures, ranging from 20 to 50°C, and it also could survive under 3% NaCl condition. These results indicated that we could optimize the fermentation conditions to promote better growth and faster enrichment of bacteria cells. Moreover, by simulating the gastrointestinal environment, we found that the strain exhibited excellent tolerance to acidity after exposure to media pH 5.0, and this indicated that *C. tyrobutyricum* L319 is highly active in the acidic gastrointestinal environment and has the potential to travel through the intestinal contents and colonize the intestinal location. Furthermore, we investigated the production of butyrate and acetate, the main components of SCFAs, under normal and acidic conditions (Figure 4). SCFAs play critical roles in regulating host metabolism, immune system, and cell proliferation. The highest concentrations of SCFAs are found in the cecum and proximal colon, where they are sources of energy for colon cells (primarily butyrate) (Koh et al., 2016). As a competitive strain for the production of SCFAs, we investigated the production of butyrate and acetate by *C. tyrobutyricum* L319, especially under acidic conditions. The results indicated that *C. tyrobutyricum* L319 has a great potential to be applied in the production of SCFAs, which could contribute to beneficial effects on human health (Figure 4; Zhu et al., 2022).

Prebiotics are substrates that are selectively utilized by the gut microbiota and promote human health (Cunningham et al., 2021). Previous studies have found that prebiotics can enhance the function of probiotics in gut microbiota and can delay the progression of immune diseases such as type 1 diabetes and acute pancreatitis by regulating the homeostasis of gut microbiota and the production of SCFAs (Fei et al., 2021). Studying the utilization of prebiotics by probiotics will contribute to the precise enrichment of target probiotics and improve their abundance. Therefore, we investigated the utilization of eight prebiotics by *C. tyrobutyricum* L319 and determined the production of butyrate and acetate. It was found that *C. tyrobutyricum* showed excellent growth performance whether using chito-oligosaccharide as a sole carbon source or using together with glucose as the mixed carbon source (Figure 5). Also, chito-oligosaccharide and glucose (1:1) mixed carbon sources were the optimal strategy for the production of SCFAs (Figure 6). Our results suggested that *C. tyrobutyricum* could ferment prebiotics, such as chito-oligosaccharides to produce SCFAs. This potential synergistic effect of prebiotics and probiotics could improve gut microbiota for health benefits.

## Conclusion

This study confirmed that *C. tyrobutyricum* L319 has no potential virulence factors or the possibility of antibiotic resistance genes propagation. It also fulfilled several criteria for probiotics, including significant hydrophobicity under acidic conditions, resistance to low and high temperatures, high salts and low pH, availability of chito-oligosaccharides, and the production of SCFAs. To the best of our knowledge, this is the first study describing *C. tyrobutyricum* strains isolated from the Grana Padano cheese food sources, which could be a promising probiotic candidate for applications. Regarding the safety and probiotic properties, this strain is expected to be developed as the first probiotics representative of *C. tyrobutyricum*. Moreover, further studies about its safety and probiotic properties *in vivo* and the optimization of prebiotics utilization and SCFAs production will be necessary to establish its actual feasibility in being used as probiotics.

## Data Availability Statement

The original contributions presented in this study are included in the article/Supplementary Material, further inquiries can be directed to the corresponding authors.

## Author Contributions

ZY performed the experiments, analyzed the data, and wrote the manuscript with the assistance of LY, LZ, YL, and ZZ. ZZ and LJ provided the experimental ideas and the design of this study. All authors edited, read, and approved the final version of the manuscript.

## Conflict of Interest

The authors declare that the research was conducted in the absence of any commercial or financial relationships that could be construed as a potential conflict of interest.

## Publisher’s Note

All claims expressed in this article are solely those of the authors and do not necessarily represent those of their affiliated organizations, or those of the publisher, the editors and the reviewers. Any product that may be evaluated in this article, or claim that may be made by its manufacturer, is not guaranteed or endorsed by the publisher.
