# Supplementary material for: Functional Characterization of Clostridium tyrobutyricum L319: A Promising Next-Generation Probiotic for Short-Chain Fatty Acid Production
Source: Front Microbiol. 2022 Jun 17;13:926710. doi: 10.3389/fmicb.2022.926710 (PMC9247582; doi:10.3389/fmicb.2022.926710)
Supplement: Supplementary file 1 [file Data_Sheet_1.docx]

***Supplementary Material***

**1. Supplementary Figures and Tables**


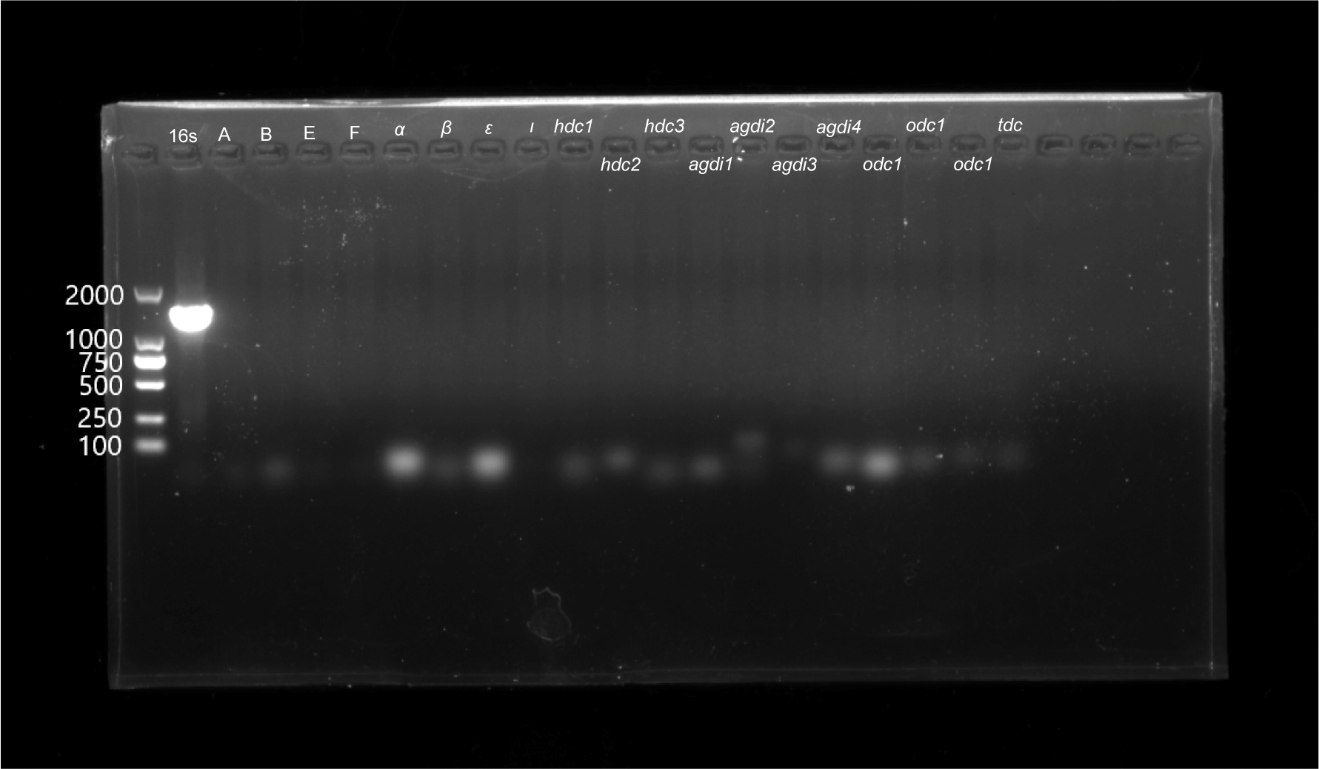


**Supplementary Figure 1.** DNA fragments obtained after PCR of the genomic DNA of *C. tyrobutyricum* with primers listed in table S2.

**Supplementary Table 1.** Distribution of different concentration of antibiotic solutions in 96-wells plate

| Antibiotics | **1** | **2**  **μg/ml** | **3**  **μg/ml** | **4**  **μg/ml** | **5**  **μg/ml** | **6**  **μg/ml** | **7**  **μg/ml** | **8**  **μg/ml** | **9**  **μg/ml** | **10**  **μg/ml** | **11**  **μg/ml** | **12** |
| --- | --- | --- | --- | --- | --- | --- | --- | --- | --- | --- | --- | --- |
| Gentamicin | P^a^ | 0.5 | 1 | 2 | 4 | 8 | 16 | 32 | 64 | 128 | 256 | N^b^ |
| Kanamycin | P | 2 | 4 | 8 | 16 | 32 | 64 | 128 | 256 | 512 | 1024 | N |
| Streptomycin | P | 0.5 | 1 | 2 | 4 | 8 | 16 | 32 | 64 | 128 | 256 | N |
| Tetracycline | P | 0.125 | 0.25 | 0.5 | 1 | 2 | 4 | 8 | 16 | 32 | 64 | N |
| Erythromycin | P | 0.016 | 0.032 | 0.063 | 0.125 | 0.25 | 0.5 | 1 | 2 | 4 | 8 | N |
| Clindamycin | P | 0.032 | 0.063 | 0.125 | 0.25 | 0.5 | 1 | 2 | 4 | 8 | 16 | N |
| Chloramphenicol | P | 0.125 | 0.25 | 0.5 | 1 | 2 | 4 | 8 | 16 | 32 | 64 | N |
| Ampicillin | P | 0.032 | 0.063 | 0.125 | 0.25 | 0.5 | 1 | 2 | 4 | 8 | 16 | N |
| Neomycin | P | 0.5 | 1 | 2 | 4 | 8 | 16 | 32 | 64 | 128 | 256 | N |
| Vancomycin | P | 0.25 | 0.5 | 1 | 2 | 4 | 8 | 16 | 32 | 64 | 128 | N |
| Linezolid | P | 0.032 | 0.064 | 0.125 | 0.25 | 0.5 | 1 | 2 | 4 | 8 | 16 | N |
| Trimethoprim | P | 0.125 | 0.25 | 0.5 | 1 | 2 | 4 | 8 | 16 | 32 | 64 | N |
| Ciprofloxacin | P | 0.25 | 0.5 | 1 | 2 | 4 | 8 | 16 | 32 | 64 | 128 | N |
| Rifampicin | P | 0.125 | 0.25 | 0.5 | 1 | 2 | 4 | 8 | 16 | 32 | 64 | N |

P^a^ was positive control, N^b^ was negative control

**Supplementary Table 2.** Positive results (+) for virulence genes, using the respective primers, in *C. tyrobutyricum* L319.

| Enzymes | *C. tyrobutyricum* L319 | Primers (5 - 3) |
| --- | --- | --- |
| BoNT/A | - | F: ATGAACCTGAAAATATTTCAATAGAAAATC  R: TTATTAACTTTATCTTTTAATCTATCTACTTG |
| BoNT/B | - | F: ATGTAGATGTTCCAGTATATGAAAAACAAC  R: ATTGTATCATTGGTATATATTGAAAGATCAAAC |
| BoNT/E | - | F: ATGAGAGATAATAATTCAGGATGGAAAGTATC  R: TGAATTCATAACTACTACTTGATTAAATCTATTG |
| BoNT/F | - | F: ATGTAGGTCTTGCTTTGAATATAATTATTG  R: ATAGTGTATTCCCGATTATGATTCATAGGTTTG |
| CPA | - | F: ATGGAAAGATTGATGGAACAGGAACTCATG  R: CAACTGATGGATCATTACCCTCTGATACATC |
| CPB | - | F: ATGCCTATTATCACCAACTTTAGTGTATGC  R: CAGCATATTCGCTTTCAATTGAAGCTTTAG |
| ETX | - | F: ATGATAATGTAGATACATTAATTGAGAAAG  R: TAAATATGCTATTACTTCTACAGTAGTATTAG |
| La/Lb | - | F: ATGGTCCTTTAAATAATCCTAATCCAGAAC  R: GTTTAGGAACAGATAAATTATCAATTGAAC |
| CbHDC | - | F: ATGATAGAGCTGAAATAAATGATGCTTATG  R: AACTGACCAAACTACTGATTTTCTATGTTC |
| CpHDC | - | F: ATGTATCAGTTCTTAAGGTTTCCGTTGGTAC  R: AAGTCCTGCATCTTCTATGAATAAATCAGC |
| CtHDC | - | F: ATGGAAATCCTCAATCTGTAGAACCAAGAG  R: TAGAACCTGCGAAGGTGCTATCTGGAGATC |
| CbeAGDI | - | F: ATGTTTGCTAAATTCTAACAGAAATCCCAAC  R: CATTACCGCCTTCTTTAATAACAGCCATTC |
| CboAGDI | - | F: ATGAGTTTAATGGCGATGGCATTATGATGG  R: CATACCATTCATATCTTCTTTTGCTTCACTCC |
| CspAGDI | - | F: ATGAGTTTAATGGTCGTGGTATTATGATGTC  R: CATACCATTCATCTCTTCTTTTGCTTCACTC |
| CsyAGDI | - | F: ATGAGAACCCTGAAGTCATGTTTGATGGAC  R: ATCATGTCCCTCGTTGAAAGGGACAATATC |
| CaODC | - | F: ATGGCAACAAAGTTTACCAATGGTTCAACC  R: TTGTTACCACCCGTTGGTAAATCACGTCTAG |
| LsODC | - | F: ATGTCTACTTCACCATTCTACCCAATGTATG  R: AAGCTTGTTGTGGTTGCGGATGAATTCTTG |
| YODC | - | F: ATGAACATCTCGCCGGATAGAATTGTTTAC  R: TCTTGTTTTGTTGATAGAGTCGAGGACCGC |
| CbTDC | - | F: ATGGTTAATACCACAAACTAAGCACTATTC  R: TCTAATTTCTTGTATAACTTATATGCACCTTC |

Abbreviation: Cb (*Clostridium baratii*); Cp (*Clostridium perfringens* ATCC 13124); Ct (*Clostridium tetani* E88); Cbe (*Clostridium beijerinckii*); Cbo (*Clostridium botulinum*); Csp (*Clostridium sporogenes*); Csy (*Clostridium symbiosum*); Ca (*C.albicans*); Ls (*Lactobacillus strain 30A*); Y (Yeast); AGDI (Agmatine deiminase); HDC (Histidine Decarboxylase); ODC (Ornithinedecarboxylase); TDC (Tyrosinedecarboxylase); BoNT (botulinum toxin); CPA (α-Toxin); CPB (β-Toxin); ETX (ε-Toxin); La/Lb (ι-Toxin)

**Supplementary Table 3** Comparison of gastrointestinal tolerance rates of different probiotic products.

| Probiotic products | gastrointestinal tolerance (%) |
| --- | --- |
| (CLOSTAT) *Bacillus subtilis* | 55.1 (Chan et al., 2010) |
| *Bacillus licheniformis* | 32.8(Bested et al., 2013) |
| *Enterococcus faecalis* | 42.0(Santos Ornellas et al., 2017) |
| *Clostridium tyrobutyricum* L319 | 69.7(This study) |

**References**

Chan ES, Lee PP, Ravindra P, Krishnaiah K, Voo WP. A standard quantitative method to measure acid tolerance of probiotic cells. Appl Microbiol Biotechnol. 2010;86(1):385-91.

Bested AC, Logan AC, Selhub EM. Intestinal microbiota, probiotics and mental health: from Metchnikoff to modern advances: Part I - autointoxication revisited. Gut Pathog. 2013;5(1):5.

Santos Ornellas RM, Santos TT, Arcucio LB, Cicco Sandes SH, Oliveira MM, Dias CV, et al. Selection of Lactic Acid Bacteria with Probiotic Potential Isolated from the Fermentation Process of "Cupuacu" (Theobroma grandiflorum). In: Donelli G, editor. Advances in Microbiology, Infectious Diseases and Public Health, Vol 7. Advances in Experimental Medicine and Biology. 9732017. p. 1-16.
